# Delayed Onset Anthracycline-Associated Cardiotoxicity Presenting as Acute Decompensated Heart Failure Seven Years After Chemotherapy Completion

**DOI:** 10.7759/cureus.16920

**Published:** 2021-08-05

**Authors:** Dan B Tran, Amro K AlAshi, Annellys Hernandez

**Affiliations:** 1 Medicine, Florida International University, Herbert Wertheim College of Medicine, Miami, USA; 2 Translational Medicine, Florida International University, Herbert Wertheim College of Medicine, Miami, USA

**Keywords:** late cardiotoxicity, anthracyclines, heart failure with reduced ejection fraction, acute decompensated heart failure, management of heart failure

## Abstract

Anthracyclines are a class of chemotherapeutic agents commonly used to treat a variety of malignancies including leukemias and lymphomas. Cardiotoxicity is a well-known clinical adverse effect of anthracyclines with a diverse range of presentations and manifestations. While the vast majority of cardiotoxicity from anthracyclines presents acutely during or within one year after chemotherapy, they rarely cause long-term effects several years after. Here, we present a case of sudden and delayed onset doxorubicin-associated cardiotoxicity seven years following chemotherapy completion. We review important and evidence-based clinical diagnostic workup and management strategies for atypical anthracycline-associated cardiotoxicity in a young patient with acute decompensated heart failure.

## Introduction

Anthracycline-associated cardiotoxicity (AAC) is a potentially fatal adverse effect of chemotherapeutic treatment of non-Hodgkin lymphomas as well several other cancers. Patients who develop heart failure as a consequence of AAC are prone to mortality rates as high as 50% [1,2]. Treating non-Hodgkin lymphomas with the combination of medications including rituximab, cyclophosphamide, Doxorubicin, Vincristine, and prednisone (R-CHOP regimen) has been shown to significantly benefit patients by potentially curing disease, decreasing the risk of relapse, and the progression of the disease [3,4]. According to the American College of Cardiology, anthracyclines are effective in treating a wide spectrum of cancers including carcinomas (breast, small cell lung, bladder, esophagus, stomach, liver, and thyroid), leukemias, lymphomas (Hodgkin’s and non-Hodgkin’s), and sarcomas (osteogenic, soft tissue, and Ewing’s) [5]. Patients who are treated with anthracyclines have approximately a 25% higher risk of developing heart failure as compared to the general population [2,5].

AAC has been widely reported in the literature and is a clinically well-known adverse effect of anthracycline-based chemotherapy. According to a 2017 study of 2625 cancer patients treated with anthracyclines, 9% developed cardiotoxicity in the form of dysrhythmias, left ventricular dysfunction, and symptomatic heart failure. Of these patients, 98% of cases developed within the first year following treatment [5]. However, only 2% had an occurrence of doxorubicin cardiotoxicity six to 10 years after treatment, indicating the predominance of acute cardiotoxic manifestations compared to long-term. Other studies showed a higher risk of acute doxorubicin-cardiotoxicity estimated at 11% compared to the risk of chronic doxorubicin-cardiotoxicity at 1.7% [6]. The risk of cardiotoxicity has a dose-dependent relationship with the anthracyclines, specifically doxorubicin [6]. Patients treated with 300 mg/m^2^ had a 1% incidence of congestive heart failure, compared to 4% in patients who received 450 mg/m^2^ of doxorubicin [1]. Doxorubicin has 10 times the affinity for cardiomyocytes, compared to other internal organs. Cardiomyocytes are terminally differentiated without regenerative capacity, therefore doxorubicin-induced oxidative stress is linked to permanent cellular damage. The pathophysiology of AAC is not completely understood but has been associated with mitochondrial swelling and dysfunction, sarcoplasmic reticulum distention, myofibrillar loss, and myocardial necrosis [7]. These cardiotoxic consequences result from doxorubicin-induced molecular alterations including increases in reactive oxygen species (ROS) within mitochondria and doxorubicin intercalation into cellular DNA resulting in DNA strand breaks, inhibition of DNA replication, and induction of cellular apoptosis [8-10]. The most common clinical manifestations of AAC include heart failure, dysrhythmias, pericarditis, myocarditis, and even asymptomatic reductions in left ventricular ejection fraction (LVEF) during or within one year after chemotherapy with anthracyclines.

In our case report, we describe an atypical presentation of AAC in a young patient manifesting for the first time in seven years since completion of R-CHOP chemotherapy as acute decompensated heart failure. We provide an overview of our clinical diagnostic evaluation as well as our management and interventional strategies for suspected AAC.

## Case presentation

**Figure 1:**
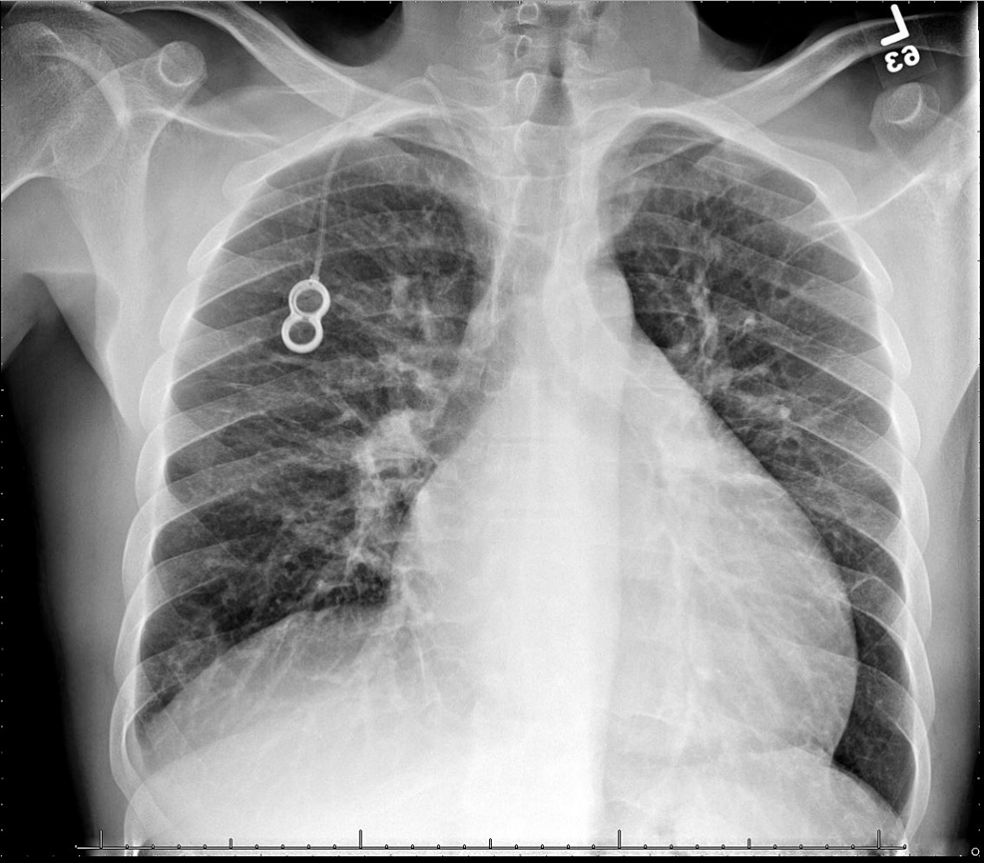
Chest x-ray on Admission Chest x-ray demonstrating cardiomegaly and interstitial pulmonary edema with cephalization.

**Figure 2:**
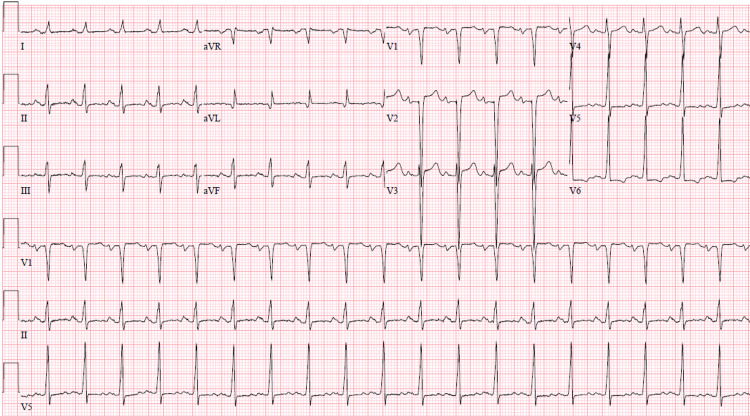
EKG on Admission 12-lead EKG on admission demonstrating sinus tachycardia and left ventricular hypertrophy with no acute ischemic ST-segment changes.

A 36-year-old male presented to the emergency department with one week of progressively worsening dyspnea at rest, fatigue, and non-productive cough. He has a past medical history significant for diffuse large B-cell lymphoma (DLBCL) of the stomach and pancreas treated with R-CHOP chemotherapy regimen in 2014, and currently in remission. Review of systems was negative for chest pain, syncope, palpitations, orthopnea, paroxysmal nocturnal dyspnea, lower extremity edema, flu-like symptoms, fever, sick contacts, recent travel, recent trauma or stressors, recent alcohol use, and positive only for recent cannabis use. Vitals were significant for tachycardia at 110 and diastolic hypertension at 138/107 mmHg. Physical examination revealed no murmurs, rubs, or gallops but was remarkable for diffuse crackles bilaterally on auscultation and jugular venous distension. Chest x-ray revealed cardiomegaly with interstitial pulmonary edema (Figure 1) and EKG was significant for sinus tachycardia with signs of left ventricular hypertrophy (Figure 2). Laboratory studies were significant for elevated D-dimer (2.48 ng/mL), elevated troponin (0.09 ng/mL), and significantly elevated N-terminal pro-B-type natriuretic peptide (NT-proBNP) (12,600 pg/mL). Computed tomography (CT) angiogram was performed, with no evidence of filling defects suggestive of pulmonary emboli. He was started on intravenous (IV) furosemide with rapid symptomatic relief and admitted to the medical floor for diagnostic workup of acute decompensated heart failure of unspecified etiology.

**Figure 3:**
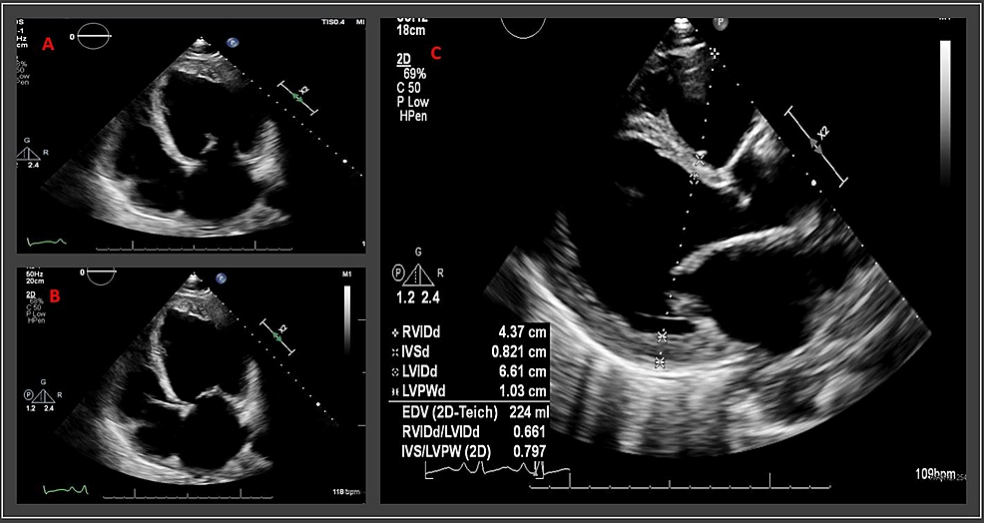
Transthoracic echocardiogram (TTE) Transthoracic echocardiogram (TTE) with apical four-chamber views in diastole (A) and systole (B) and parasternal long-axis view (C) demonstrated global left ventricular hypokinesis and markedly reduced left ventricular ejection fraction (EF) of 10-15%.

Transthoracic echocardiogram (TTE) was performed and revealed global left ventricular (LV) wall hypokinesis with LV ejection fraction (EF) of 10-15% as well as an LV thrombus (Figures 3A-3C). For assessment of an ischemic etiology, cardiac catheterization was performed with coronary angiography which revealed non-ischemic cardiomyopathy (NICMP), non-obstructive coronary artery disease, and normal pulmonary pressures. Viral pathogen panels, including HIV, influenza A and B, and SARS-CoV-2, were negative for an infectious etiology. IgG titers for *Trypanosoma cruzi* were not detected. Thyroid-stimulating hormone (TSH) levels for evaluation of thyroid-induced cardiomyopathy were within normal limits (2.78 mIU/mL). Urinalysis was unremarkable and urine toxicology screen was positive only for cannabinoids which were confirmed by our patient. CT scan of the abdomen and pelvis was unremarkable and negative for recurrence of malignancy. Further review of our patient’s medical records confirmed his completion of seven cycles of rituximab, cyclophosphamide, doxorubicin, vincristine, and prednisone (R-CHOP) for treatment of DLBCL in 2014, and that his TTE prior to chemotherapy was within normal limits (EF 55-60%), without a repeat TTE until this time.

As a diagnosis of exclusion, our patient was diagnosed with acute decompensated heart failure secondary to a delayed doxorubicin-induced, non-ischemic cardiomyopathy. He was initiated on a heparin drip for treatment of his LV thrombus and guideline-directed medical therapy (GDMT) for heart failure with reduced ejection fraction (HFrEF) including sacubitril-valsartan and spironolactone with the addition of metoprolol succinate and dapagliflozin scheduled for discharge. On day four of hospitalization, our patient was euvolemic with significant symptomatic improvement. He was discharged home with his regimen of GDMT for HFrEF, oral furosemide, apixaban for anticoagulation, a LifeVest wearable cardioverter-defibrillator for sudden cardiac death prevention and was scheduled for outpatient cardiology follow-up.

## Discussion

Anthracyclines are a class of chemotherapeutic medications commonly used in the treatment of non-Hodgkin lymphomas and include agents such as doxorubicin, daunorubicin, and idarubicin. One of the most well-known adverse effects of anthracyclines is cardiotoxicity involving both early and late clinical manifestations such as heart failure, dysrhythmias, pericarditis, myocarditis, and even asymptomatic reductions in LVEF. The prevalence of anthracycline-associated cardiotoxicity (AAC) is poorly defined due to the lack of standardized diagnostic criteria as well as the diversity of manifestations depending on the cumulative dose of anthracyclines received [1]. However, current estimates of cardiotoxicity incidence range between 5% and 45% with the vast majority of cases presenting either acutely during chemotherapy or within two to three years following chemotherapy completion [2]. Here, we presented an unusual and atypical presentation of a delayed, yet sudden, onset of AAC in a young patient over seven years following chemotherapy treatment for DLBCL as well as our clinical diagnostic and management strategy for doxorubicin-induced acute heart failure.

Known risk factors for the development of AAC include age over 50 years, obesity, preexisting cardiovascular disease, hyperlipidemia, and diabetes mellitus - none of which were present in our patient [9]. While AAC was included in our differential diagnosis upon admission, more common etiologies were pursued during cardiac workup according to evidence-based guidelines given how the onset of cardiotoxicity over seven years following chemotherapy completion has been rarely reported in the literature. Following confirmation of heart failure via echocardiography and elevated NT-proBNP, careful history and physical examination guided our risk factor assessment for cardiomyopathy from alternative etiologies. This included the absence of flu-like symptoms or sick contacts for viral myocarditis, absence of recent travel for *Trypanosoma cruzi* or Chagas disease, minimal consumption of alcohol for alcoholic cardiomyopathy, negative review of systems for thyroid-induced cardiomyopathy, and negative coronavirus disease 2019 (COVID-19) vaccination status for rare incidences of post-vaccination carditis. These hypotheses were evaluated via laboratory testing as described in the case presentation, which ruled out these alternatives. However, cannabis use was considered as a possible rare etiology of acute heart failure, a review of the literature did not elucidate a compelling body of evidence to support the association, while multiple studies have long established prior anthracycline exposure and its pathophysiology as a stronger and more likely risk factor for cardiac pathology. In accordance with guideline-directed heart failure workup, evaluation for ischemic cardiomyopathy was performed via cardiac catheterization and coronary angiography which effectively excluded obstructive coronary artery disease (CAD). While he did not have risk factors for ischemic cardiomyopathy, such as diabetes, hyperlipidemia, hypertension, history of anginal chest pains, or obesity, evaluation for obstructive CAD is usually indicated in the workup for acute heart failure of unknown etiology. Therefore, delayed onset AAC was our presumed diagnosis after evidence-based exclusion of alternative etiologies and his primary risk factor of cumulative doxorubicin exposure years prior to his acute onset of symptoms.

Empiric management for our patient’s symptoms and underlying cardiovascular pathology was initiated concurrently with our diagnostic workup. Intravenous furosemide was provided for rapid symptomatic relief while laboratory studies and rule-out of pulmonary emboli were performed, given the elevated D-dimer. Following elucidation of heart failure with reduced ejection fraction (HFrEF) of 10-15%, guideline-directed medical therapy (GDMT) was initiated. However, there are limited studies in the literature describing the use or overall efficacy of HFrEF GDMT specifically in patients with AAC [1]. He was thus empirically treated with sacubitril-valsartan and spironolactone for evidence-based optimization of cardiac function and prolongation of survival while heparin was started for treatment of the incidental LV thrombus. While close outpatient follow-up with cardiovascular specialists and initiation with a beta-adrenergic antagonist and sodium-glucose-cotransporter-2 inhibitor (SGLT2i) were planned upon medical stabilization and discharge, long-term prognosis of AAC is not well understood [1]. While our patient was discharged with planned outpatient follow-up with cardiology, cardiac magnetic resonance imaging (CMR) for a more detailed evaluation of his cardiac structure and function may further aid in confirming the diagnosis. Importantly, we highlight in our report an unusual and diverse presentation of the long-term effects of anthracycline-based chemotherapy as well as the need for further research on strategies for routine monitoring and prevention of cardiotoxic manifestations of anthracyclines years after administration. Risk factor assessment, thorough history-taking, and evaluation of the degree of chemotherapy exposure may aid clinicians in suspecting atypical late manifestations of anthracycline-associated cardiotoxicity. Evidence-based and guideline-directed cardiac workup and medical therapy should still form the standard of care for post-chemotherapy patients presenting with acute decompensated heart failure.

## Conclusions

In our case report, we present an atypical presentation of delayed onset anthracycline-associated cardiotoxicity (AAC) as well as our extensive workup of differential diagnoses of acute heart failure. Long-term manifestations of anthracycline-associated adverse cardiotoxic effects are not well understood and potentially underreported. Here, we emphasized and summarized the appropriate evidence-based and guideline-directed diagnostic and management strategies for acute decompensated heart failure of undetermined etiology as well as establishing a diagnosis of exclusion for AAC.
